# Culturable associated-bacteria of the sponge *Theonella swinhoei* show tolerance to high arsenic concentrations

**DOI:** 10.3389/fmicb.2015.00154

**Published:** 2015-02-25

**Authors:** Ray Keren, Adi Lavy, Boaz Mayzel, Micha Ilan

**Affiliations:** Department of Zoology, George S. Wise Faculty of Life Sciences, Tel Aviv UniversityTel Aviv, Israel

**Keywords:** arsenic, Porifera, symbionts, bacterial cultivation, *Theonella swinhoei*, bioremediation

## Abstract

Sponges are potent filter feeders and as such are exposed to high fluxes of toxic trace elements, which can accumulate in their body over time. Such is the case of the Red Sea sponge *Theonella swinhoei*, which has been shown to accumulate up to 8500 mg/Kg of the highly toxicelement arsenic. *T. swinhoei* is known to harbor a multitude of sponge-associated bacteria, so it is hypothesized that the associated-bacteria will be tolerant to high arsenic concentration. This study also investigates the fate of the arsenic accumulated in the sponge to test if the associated-bacteria have an important role in the arsenic accumulation process of their host, since bacteria are key players in the natural arsenic cycle. Separation of the sponge to sponge cells and bacteria enriched fractions showed that arsenic is accumulated by the bacteria. Sponge-associated, arsenic-tolerant bacteria were cultured in the presence of 5 mM of either arsenate or arsenite (equivalent to 6150 mg/Kg arsenic, dry weight). The 54 isolated bacteria were grouped to 15 operational taxonomic units (OTUs) and isolates belonging to 12 OTUs were assessed for tolerance to arsenate at increased concentrations up to 100 mM. Eight of the 12 OTUs tolerated an order of magnitude increase in the concentration of arsenate, and some exhibited external biomineralization of arsenic–magnesium salts. The biomineralization of this unique mineral was directly observed in bacteria for the first time. These results may provide an explanation for the ability of the sponge to accumulate considerable amounts of arsenic. Furthermore arsenic-mineralizing bacteria can potentially be used for the study of bioremediation, as arsenic toxicity affects millions of people worldwide.

## INTRODUCTION

Sponges (phylum Porifera) are evolutionarily ancient metazoans, populating all aquatic habitats (Taylor et al., 2007). They are filter-feeders that can filter up to 50,000 times their body volume in 24 h (Weisz et al., 2008). The high water volume passing through sponges from the surrounding environment exposes them to high amounts of toxic elements, even if these are present only at trace concentrations (Mayzel et al., 2014). Combined with their important role in establishing benthic communities, sponges offer a good tool for biomonitoring ecosystem health (Maldonado et al., 2012).

The coral reef sponge *Theonella swinhoei* harbors a dense consortium of associated-bacteria, which occupy up to 40% of its body volume (Hentschel et al., 2006; Taylor et al., 2007), reaching up to 10^10^ bacteria/ml sponge (Gloeckner et al., 2014). *T. swinhoei* is common in the Indo-Pacific Ocean and its extension, the Red Sea (Ilan et al., 2004). Some of the bacteria inhabiting *T. swinhoei* are photosynthetic while others are heterotrophic. Most of the bacteria are unicellular but there are also filamentous bacteria (*Entotheonella* sp.; Magnino et al., 1999; Hentschel et al., 2002; Schmitt et al., 2012). Several studies utilizing culture-independent techniques, identified within *T. swinhoei* over 100 bacterial operational taxonomic units (OTUs), mainly of the phyla *Proteobacteria*, *Chloroflexi*, *Poribacteria*, *Acidobacteria*, *Actinobacteria*, and *Cyanobacteria* (Hentschel et al., 2002; Schmitt et al., 2012). A culture-based study of *T. swinhoei* associated-bacteria identified a diverse bacterial community, with some of its members growing under microaerophilic conditions (Lavy et al., 2014). Bacteria were cultured under one or more of 48 different treatments, differing by composition, oxygen levels, and supplementation of antibiotics. Many of the bacteria only grew under a single set of conditions, implying specific metabolic demands of their populations.

Trace elements analyses of Red Sea sponges in the Gulf of Aqaba demonstrated that *T. swinhoei* is an accumulator of arsenic and barium (Mayzel et al., 2014). Arsenic concentration within *T. swinhoei* averaged 8500 mg/Kg (dry weight), compared to 0.003 mg/Kg in ambient seawater (personal Communication, Dr. Shaked, Head of National Monitoring program, Gulf of Aqaba). These concentrations are the highest ever recorded in an organism from an uncontaminated environment (Gibbs et al., 1983). Barium, linked to the sulfur cycle (Derry and Murray, 2004) and carbon cycle (González-Muñoz et al., 2003), was also found at extremely high concentrations in *T. swinhoei*, reaching over 10,000 mg/Kg (dry weight). Applying Principal Coordinates (PCO) analysis, it was shown that both elements are actively taken up by the sponge (Mayzel et al., 2014). *T. swinhoei* is thus an arsenic hyper-accumulating organism, similar to the fern *Pteris vittata* (Ma et al., 2001), and the polychaete *Tharyx marioni* (Gibbs et al., 1983).

Arsenic is a ubiquitous and extremely toxic element found in aquatic environments, in soils and sediments, and in organisms (Cullen and Reimer, 1989). Arsenic is also mobilized in the environment by biological activities, mainly by bacteria that solubilize arsenic from pyrites and other arsenic-containing ores (Oremland and Stolz, 2003). Bacteria play a crucial role in the arsenic cycle, modifying arsenic in various ways, such as reduction/oxidation reactions and assimilation into organic material (Stolz and Oremland, 1999; Oremland and Stolz, 2003). Moreover, in some bacteria both the reduction and oxidation (redox) of arsenic can occur as part of the respiratory electron transfer (Dowdle et al., 1996; Stolz and Oremland, 1999; Oremland and Stolz, 2003).

Most studies have focused on arsenic content in higher metazoans (Maher, 1984, 1985; Larsen et al., 1997; Kubota et al., 2001, 2002; Kirby and Maher, 2002; Casado-Martinez et al., 2010, 2012), seaweed and alga (Morita and Shibata, 1987; Shibata and Morita, 1989) while only two studies examined sponges (Yamaoka et al., 2001, 2006). These latter studies showed that some sponges (a *Theonella* species among them) contain high levels of water-soluble arsenic, although nothing as high as in *T. swinhoei* from the Gulf of Aqaba. Those studies found arsenobetaine to be the dominant arsenic species, and concluded that it was produced by the sponges themselves and not by their symbionts (Yamaoka et al., 2001, 2006). A closer examination of these results indicates that in some species arsenosugar attributed to *Cyanobacteria* is more dominant, whereas in others the dominant arsenic form was not identified. No study to date has investigated the role that associated-bacteria of invertebrates play in the response mechanism to arsenic.

To test the hypothesis that *T. swinhoei* harbors associated-bacteria that contribute to the accumulation process of arsenic and barium in their host, the concentration of these elements was measured and compared in sponge-cells-enriched and bacteria-enriched fractions. Additionally, the high arsenic concentration within the sponge led to the hypothesis that sponge-associated bacteria would be tolerant to arsenic. To that end, sponge-derived bacteria were inoculated onto culturing media containing either arsenate or arsenite, in order to investigate the bacterial community that may survive and even thrive in the presence of this highly toxic element.

## MATERIALS AND METHODS

### SAMPLING AND PROCESSING OF SPONGES

*Theonella swinhoei* samples (*n* = 4) were collected at Eilat, Red Sea (N 34^∘^55^′^02^′′^/E 29^∘^60^′^05^′′^) by SCUBA diving at 15–20 m depth and processed on site at the Interuniversity Institute for Marine Sciences. All work henceforth was performed in a laminar flow hood under sterile conditions. Sponges were thoroughly rinsed in sterile calcium–magnesium–free artificial seawater (CMF-ASW; Strathmann, 1987) to remove transient bacteria, followed by homogenization through a juicer (Moulinex, France) with CMF-ASW. The resulting homogenate was repetitively agitated by stirring, to separate clumps of cells. Sponge cells were separated by passive settling and filtration through a 50 μm pore nylon mesh (Sc fraction). The supernatant was centrifuged at 3000 *g* to enrich the pellet for bacteria (Bac fraction). Microscopic examination validated that the bacteria fraction was clean of sponge cells but not vice versa.

For elemental analyses, supernatant was removed from all samples and pellets were kept at -20^∘^C, followed by lyophilization. For culturing, an aliquot of the bacteria pellet from one of the sponges was re-suspended in CMF-ASW and serially diluted (×10) five times. All types of culturing media were inoculated with 100 μl of each dilution in duplicate. All dilutions were used for aerophilic conditions, while only the 1:100 and 1:1000 dilutions were used for microaerophilic conditions. Plates were sealed with Parafilm and incubated at 25^∘^C. Colony formation was observed for 2 months and each new colony was re-inoculated onto fresh plates until pure isolates were obtained. Plates were routinely checked for signs of drying, whereupon colonies were restreaked onto fresh plates.

### ELEMENTAL ANALYSIS OF CELL FRACTIONS

Analyses of total element concentration were performed with inductively coupled plasma atomic emission spectroscopy (ICP-AES), using Spectro “ARCOS-SOP” (Spectro GMBH, Kleve, Germany) at the Hebrew University of Jerusalem. Acid digestion of samples followed EPA method 3050B [United States Environmental Protection Agency (USEPA), 1982] described in a prior work (Mayzel et al., 2014).

Statistical analysis was performed using R statistics (R Development Core Team, 2012) with RStudio IDE (RStudio, USA). Significant values for all tests were regarded for *p* < 0.05. Sample means were checked for significant differences between fractions by one-way ANOVA with permutations, using the lmPerm (Wheeler, 2010) package, and Tukey *post hoc* test to assign groups. The analyzed samples contained four observations per fraction, for which the required assumptions for parametric tests could not be ascertained. Permutations enabled the use of the stronger parametric tests using the true distribution and homogeneity of the samples. Differences were checked for each element separately.

### CULTURING MEDIA PREPARATION

Selective media in this study were designed in a modular fashion (**Table 1**), each type of medium designed to select for different arsenic modifying bacteria. Media composition and concentration of arsenic (5 mM of either arsenate or arsenite) were selected according to the *in vivo* measurements conducted in the present and a former work on *T. swinhoei* (Mayzel et al., 2014), as well as a literature review of similar works (Hoeft et al., 2004; Fan et al., 2008; Liao et al., 2011 ), and an expert advice (pers. comm. Prof. Oremland, USGS).

**Table 1:** Selective conditions applied in modular media design.

Medium name	Arsenic form	Aeration	Supplemented carbon source	Additional nutrient supplemented
AsVOC	Arsenate	Aerobic	Carbohydrates mix	None
AsVanC	Arsenate	Microaerophilic	Carbohydrates mix	None
AsVanS	Arsenate	Microaerophilic	Bicarbonate	Sulfide
AsIIIOC	Arsenite	Aerobic	Carbohydrates mix	Nitrate
AsIIIanC	Arsenite	Microaerophilic	Carbohydrates mix	Nitrate
AsIIIanN	Arsenite	Microaerophilic	Bicarbonate	Nitrate
AsIIIanP	Arsenite	Microaerophilic	Bicarbonate	None

Since both arsenate and arsenite enter the cells passively (Slyemi and Bonnefoy, 2012) and induce cytotoxic damage, all isolates growing on media supplemented with arsenic are considered in this work as arsenic-tolerant bacteria. Arsenate is an antagonist of phosphate (Slyemi and Bonnefoy, 2012) therefore inorganic phosphate in the media was limited to ensure that arsenate to phosphate ratio be in favor of arsenate. The final concentration of phosphate in the media was 3.5 μg/L, found in the ASW (manufacture manual, Red Sea, Israel). Thus the As:Pi ratio was approximately 20,000:1.

Basal medium consisted of 35 ppt artificial sea salt (Red Sea, Israel), BaCl_2_ (5 mM), 1 ml of vitamin solution (biotin 2 mg/l, folic acid 2 mg/l, pyridoxine-HCl 10 mg/l, thiamine HCl 5 mg/l, riboflavin 5 mg/l, nicotinic acid 5 mg/l, D-Ca-pantothenate 5 mg/l, β-cyano L-alanine 0.1 mg/l, *P*-aminobenzoic acid 5 mg/l, lipoic acid 0.5 mg/l) and 20 g of bacteriological agar. Arsenic was added as either arsenate or arsenite, at a final concentration of 5 mM. The arsenic concentration is equivalent to 6150 mg/Kg (dry weight). Calculation of arsenic concentration is as follows; both arsenate and arsenite contain a single atom of arsenic, so the arsenic concentration is 5 mM, or 375 mg/L. Each medium contains 61 g (0.061 kg) of added salts, minerals and agar. Dividing the amount of arsenic by the total amount of dry material in the medium gives the arsenic concentration of 6150 mg/Kg.

The supplemented organic carbon source in the media was a mix of Na-pyrovate, Na-acetate, and Succinic acid (1.7 g/l each). Inorganic carbon source was NaHCO_3_ (5 g/l). Bacteriological agar contains organic impurities so all media can support heterotrophic bacterial growth. NaNO_3_ (5 mM) was added, as electron acceptor, to media supplemented with arsenite (with the exception of AsIIIanP). Na_2_S (5 mM) was added, as electron donor, to medium AsVanS.

Media supplemented with carbohydrates and kept in aerobic conditions (AsVOC, AsIIIOC) were designed to select for arsenic-resistant bacteria. Media under microaerophilic conditions (AsVanC, AsVanS, AsIIIanC, and AsIIIanN) were designed to select for dissimilatory arsenate reduction and chemolithotrophic arsenite oxidation, respectively. Medium AsIIIanP that was not supplemented with any electron acceptors and was designed to select for anoxygenic photosynthesis by exposing the plates to natural sunlight. Microaerophilic work was done in a glove-box (Shel Lab, USA), under positive atmospheric pressure with nitrogen gas. Prior to each work session, the glove box was flushed with nitrogen gas three times. Microaerophilic conditions, for long term plate incubation, were maintained by placing oxygen-absorbing sachet BD GasPak^TM^ (BD, NJ, USA) and colorimetric indicator in sealed boxes.

### MOLECULAR IDENTIFICATION OF ISOLATES AND PHYLOGENETIC ANALYSIS

A single colony from each isolate was sampled into 50 μl of ultra-pure water and lysed by two cycles of heating to 95^∘^C for 5 min, followed by 1 h at -80^∘^C. 16S rRNA gene was amplified using universal bacterial primers 63f and 1387r (Marchesi et al., 1998). PCR profile was as follows: 5 min. denaturation at 94^∘^C, followed by 30 cycles of 1 min. denaturation at 94^∘^C, 1 min. 20 s. annealing at 54^∘^C, and 2 min. elongation at 72^∘^C, ending with 10 min. elongation at 72^∘^C. PCR products were sequenced at MCLAB (MCLAB, CA, USA) using primer 63f.

Obtained sequences were manually edited in FinchTV© (Geospiza, WA, USA) to remove low quality nucleotide readings. Short sequences (<600 bp) were removed from the database. Next, sequences were submitted to the Ribosomal Database Project (RDP) for classification (genus level) and alignment (Cole et al., 2007, 2009; Wang et al., 2007). Aligned sequences were clustered to OTUs at 97% similarity by Mothur software (Schloss et al., 2009), and labeled TSASRA001–TSARA102 (a representative sequence of each OTU was submitted to Genbank; KJ573540–KJ573571). The obtained OTUs were compared to known type strains using EzTaxon-e server (Kim et al., 2012).

### ARSENIC TOLERANCE ASSAY

A growth assay on solid media was chosen as a first screen to test the tolerance of bacteria to arsenate. Optical density based tests in liquid media were disqualified, as the media were opaque. A double-blind tolerance assay was conducted on gridded plates with the following concentrations of AsV: 0 mM, 10 mM, 20 mM, 50 mM and 100 mM. The two media used for the assay were AsVOC and AsVanS.

Isolates in this assay were chosen after initial molecular identification and prior to grouping into OTUs. Isolates belonging to Proteobacteria (classes *Alpha*- and *Gamma-proteobateria*), growing on media supplemented with arsenate were selected for the assay, since they composed the majority of the isolated bacteria and had members growing on both arsenate supplemented media.

A single colony of each isolate was suspended in 50 μl of CMF–ASW and plates were inoculated with 2 μl of suspension in triplicates. Each repeat was placed on a separate plate in random order. CMF–ASW without bacteria was used as a negative control. Growth was recorded after 7 days by two observers (separately) and ranked from zero to three, according to the number of repeats that grew. When an OTU was represented by more than one isolate, the growth rank average was calculated. OTUs with a growth rank higher than one are considered to be positively tolerant to the arsenate concentration examined. OTUs with an average growth rank of one were considered negative since they may have benefited from arsenate detoxification by nearby isolates. OTUs ranked less than one were considered negatively affected. Taxonomic identity of isolates was verified by re-streaking bacteria onto a fresh plate to test for purity and sequencing their 16S rRNA gene (as described above).

### ELEMENT ANALYSIS BY SCANNING ELECTRON MICROSCOPE ENERGY-DISPERSIVE X-RAY SPECTROSCOPY (SEM-EDX)

Isolates that showed precipitation in the arsenic tolerance assay, were further analyzed for elemental content by a JSM-3600 SEM-EDX (JEOL, Japan). Colonies were fixed in 2.5% glutaraldehyde (ASW), followed by serial dehydration steps in ethanol and finally air-dried with nitrogen gas flow. Dry samples were coated with palladium. Results are given in relative ratio of elements mass (element%) and the relative ratio of elements atoms (atom%).

## RESULTS

### ANALYSIS OF ARSENIC AND BARIUM IN CELL FRACTIONS

**FIGURE 1:**
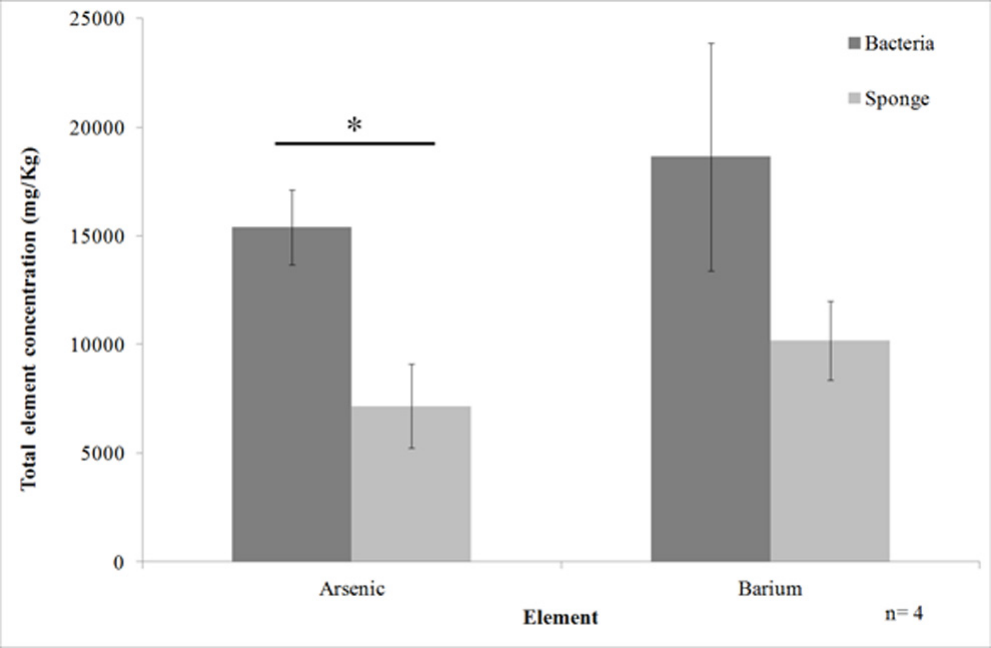
**Arsenic and barium concentration in the cell enriched fractions of *Theonella swinhoei* (Mean ± SE).** Line with asterisk above bars indicates significant difference in concentration between cellular fractions.

Element analysis of the cell fractions of *T. swinhoei* showed that the majority of arsenic was located in the bacteria-enriched fraction. Arsenic concentration in the bacteria-enriched fraction averaged 15400 mg/Kg (SE ± 1750) compared to 7200 mg/Kg (SE ± 1930) in the sponge-enriched fraction (**Figure 1**). This difference was shown to be significant (ANOVA, *n* = 4, *F* = 10, *df* = 6, *p* < 0.0195). Barium concentration in the bacteria-enriched fraction was also higher than in the sponge-enriched fraction (average of 18636 mg/Kg, compared to 10182 mg/Kg), but the difference was not statistically significant.

### BACTERIA GROWTH AND IDENTIFICATION

Bacteria grew on four of the seven designed arsenic-rich media (AsVOC, AsVanS, AsIIIanN, and AsIIIanC). Most of the bacteria isolated in this work grew on medium AsVOC (38 isolates), followed by AsVanS (13 isolates). Only three isolates grew on media supplemented with arsenite. In total, 54 isolates of arsenic-tolerant bacteria, grouped into 15 OTUs, were described. The OTUs are of the classes *Actinobacteria*, *Alphaproteobacteria*, or *Gammaproteobacteria* and comprise eight genera (**Table 2**).

**Table 2:** Phylogenetic identification of operational taxonomic units (OTUs) isolated from *Theonella swinhoei* to closest type strain in EzTaxon.

Media	OTUs	Closest type strain	Similarity to type strain	Number of isolates
AsVOC	TSASRA030	*Brevibacterium casei* DSM20657(T)	0.994	1
	TSASRA059	*Micrococcus yunnanensis* YIM65004(T)	0.992	1
	TSASRA006	*Ruegeria arenilitoris* G-M8(T)	0.995	4
	TSASRA003	*R. conchae* TW15(T)	0.992	27
	TSASRA018	*R. lacuscaerulensis* ITI-1157(T)	0.972	2
	TSASRA029	*R. scottomollicae* LMG 24367(T)	0.979	1
	TSASRA037	*Vibrio harveyi* ATCC 14126(T)	0.989	1
	TSASRA026	*V. owensii* DY05(T)	0.988	1
AsVanS	TSASRA003	*R. conchae* TW15(T)	0.992	5
	TSASRA084	*Aliagarivorans taiwanensis* AAT1(T)	0.981	1
	TSASRA002	*Pseudoalteromonas mariniglutinosa* KMM3635(T)	0.991	1
	TSASRA007	*Pseudomonas oleovorans* subsp. lubricantis RS1(T)	0.999	2
	TSASRA054	*Pseudomonas xanthomarina* KMM1447(T)	0.972	1
	TSASRA037	*V. harveyi* ATCC 14126(T)	0.989	1
	TSASRA072	*V. mediterranei* CIP10320(T)	0.999	1
	TSASRA019	*V. maritimus* R-40493(T)	0.992	1
AsIIIOC	TSASRA102	*Endozoicomonas elysicola* MKT110(T)	0.998	1
AsIIIanN	TSASRA019	*V. maritimus* R-40493(T)	0.992	2

Three of the 15 OTUs grew on two media types, and 12 were observed only on a single medium. 14 OTUs (51 isolates) grew in the presence of arsenate, while only two OTUs (three isolates) were isolated in the presence of arsenite. The same number of OTUs was isolated from aerobic conditions with carbohydrates as under microaerophilic conditions with bicarbonate, but the number of isolates differed (39 vs. 15, respectively). Since bacteriological agar contains minute impurities of organic material, all OTUs are regarded as heterotrophs.

The two *Actinobacteria* OTUs grew only under aerobic conditions, with supplemented carbohydrates and arsenate. All *Alphaproteobacteria* isolates were of the genus *Ruegeria* and grouped to four OTUs. Three of the four *Ruegeria* OTUs, grew only under aerobic conditions with supplemented carbohydrates and arsenate. TSASRA003 was the only OTU of this genus to additionally grow under microaerophilic conditions, with no added organic carbon. TSASRA003 was the most abundant OTU, with 27 isolates from medium AsVOC and five isolates from AsVanS. Gammaproteobacteria was the most diverse class, with five genera (*Aliagarivorans, Endozoicomonas, Pseudoalteromonas, Pseudomonas*, and *Vibrio*) and nine OTUs. *Gammaproteobacteria* isolates grew under all culture conditions, and were more abundant than *Alphaproteobacteria* under microaerophilic conditions. Moreover, only *Gammaproteobacteria* isolates grew in the presence of arsenite. Among the *Gammaproteobacteria*, the genus *Vibrio* was the most resilient, growing on three out of four media (AsVOC, AsVanS, and AsIIIanN).

A BLAST search against NCBI database Nucleotide collection with the OTU sequences was conducted with two Entrez Query (“sponge” and “*Theonella*”). Bacteria previously isolated by our group (Lavy et al., 2014) were disregarded in the search. With the “sponge,” query, 14 of 15 OTUs had hits with >97% sequence similarity. For TSASRA084 a hit with 94% sequence similarity was found. The last is a recently discovered genus (Jean et al., 2009). With the “*Theonella*” query, five OTUs had hits with >97% sequence similarity. Eight OTUs had hits at the genus level (92–96%) and an additional two OTUs had hits at the family level (88–89%), among them TSASRA084.

### ARSENATE-TOLERANCE ASSAY

**FIGURE 2:**
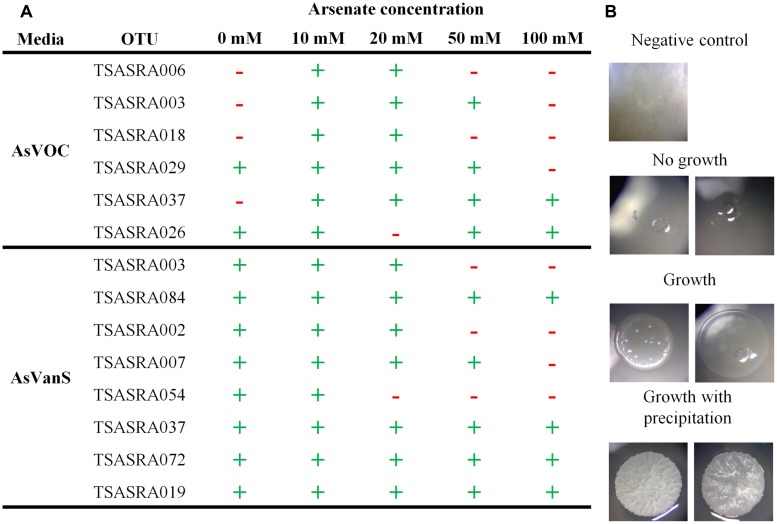
**Tolerance of isolated operational taxonomic units (OTUs) to increased or decreased arsenate concentration. (A)** Observation of OTU growth at different arsenate concentration. (**+**) marks growth and (**–**) marks no growth. **(B)** Representative image of isolates observed to grow (with and without precipitation) or not.

The majority of OTUs (eight of 12) tolerated an increase of arsenate concentration from 5 to 50 mM (**Figure 2A**). Moreover, four of those eight arsenate-tolerant OTUs grew on 100 mM arsenate. Such arsenic concentration (123000 mg/Kg, dry weight) is an order of magnitude higher than found in the bacterial enriched fraction (**Figure 1**). Growth was highest at 10 mM, on which all OTUs grew, and 20 mM where all but one OTU grew.

The isolates were also assayed for growth without addition of arsenate to the media (**Figure 2A**). Six OTUs were tested under aerobic conditions with supplemented carbohydrates in the medium (AsVOC). Only two of the six OTUs grew without arsenate present. In contrast, all OTUs grew without arsenate under microaerophilic conditions with supplemented bicarbonate and sulfide in the medium (AsVanS). This is especially notable for TSASRA003 and TSASRA037, the only OTUs growing on both media. Both did not grow on AsVOC at 0 mM arsenate but did grow on AsVanS, without arsenate in the medium. On the other hand, TSASRA003 tolerated up to 50 mM arsenate on AsVOC and only 20 mM arsenate on AsVanS.

### PRECIPITATION OF ARSENIC

**FIGURE 3:**
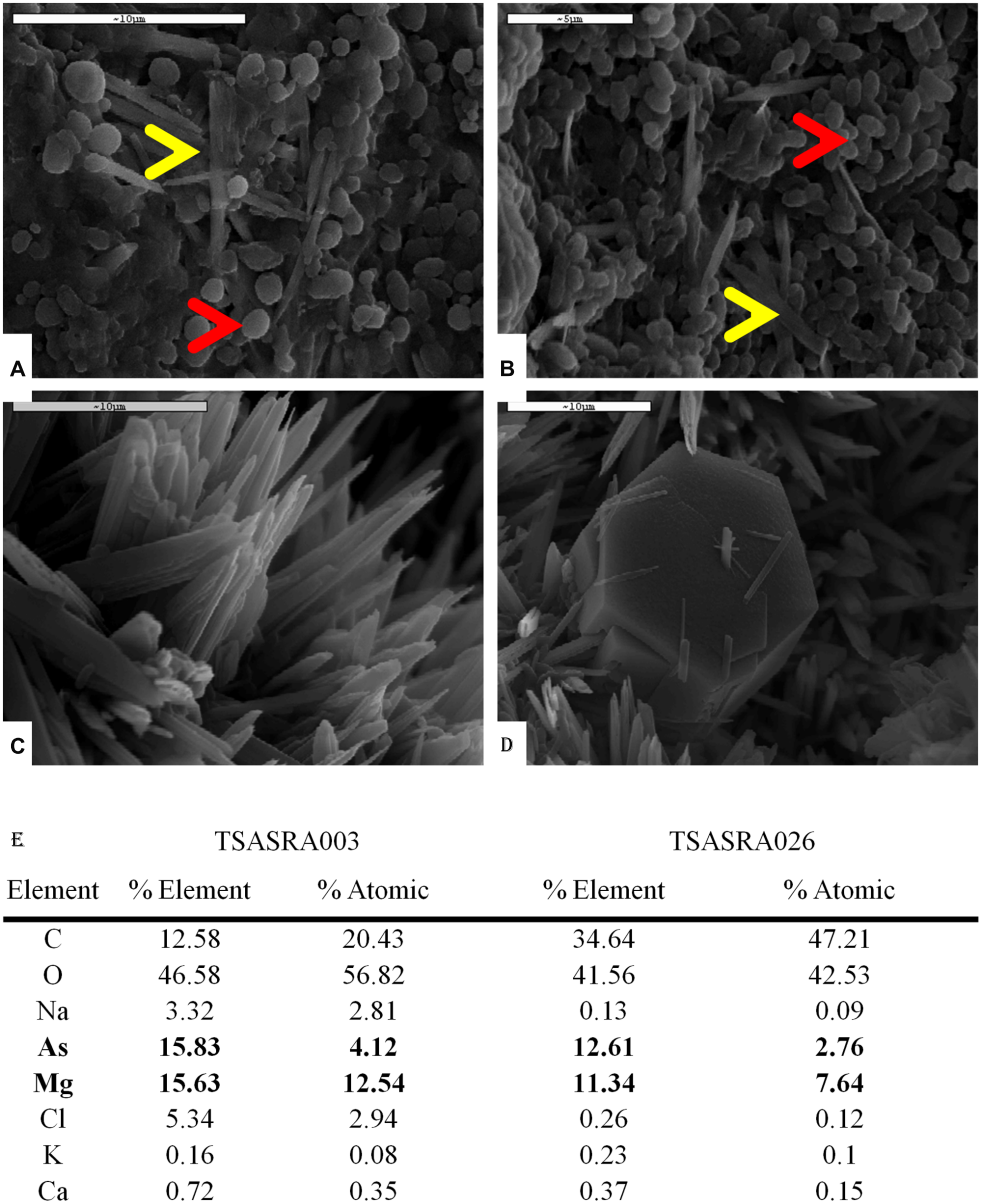
***Theonella swinhoei* isolated bacteria precipitating arsenic salt.** All SEM images were taken at 10 Ev and element analysis conducted at 20 Ev. EDX analysis results are presented as %Element showing the relative mass of the element, and %Atomic showing the relative amount of the element. **(A)** TSASRA003- Bacteria at border of the colony (red arrow) with needle-like crystals (yellow arrow). **(B)** TSASRA026- Bacteria at border of the colony (red arrow) with needle-like crystals (yellow arrow). **(C)** Needle-like crystals. **(D)** Secondary crystal (hexagonal), surrounded by Needle-like crystals. **(E)** EDX analysis showing elevated As and Mg in both bacteria.

During the arsenic tolerance assay precipitation was observed in 18 isolates, at 10–20 mM arsenate (**Figure 2B**). Several isolates, grouped into two OTUs, externally precipitated arsenic, as observed under SEM-EDX examination (**Figure 3**). In the negative control no arsenic was detected, indicating free arsenate was washed away during the sample preparation.

The arsenic precipitating OTUs were identified as TSASRA003 (**Figure 3A**) and TSASRA026 (**Figure 3B**). The colony of TSASRA003 was completely covered by a layer of precipitate. SEM image of the colony center showed a dense mineral layer with bacterial cells only noticeable at the periphery (**Figure 3A**, red arrow). The mineral covering the colony had a needle-like morphology (**Figure 3A**, yellow arrow and **Figure 3C**). The colony of TSASRA026 was also fully covered by precipitate but had a glassy veneer. Similar to TSASRA003, bacterial cells were only noticeable at the periphery of the colony (**Figure 3B**, red arrow). The colony’s center portrayed the same dense coverage of the needle-like crystal as the major mineral (**Figure 3B**, yellow arrow), though an additional second hexagonal mineral was also observed (**Figure 3D**). The secondary mineral had higher levels of sulfur and calcium (data not shown). Although the bacteria have different taxonomic identification (**Table 2**), both precipitated the same needle-like mineral crystals (**Figure 3C**) enriched for arsenic and magnesium (**Figure 3E**), at a ratio approximately 1:3 (As:Mg, Atomic%).

## DISCUSSION

### ANALYSIS OF ARSENIC AND BARIUM IN CELL FRACTIONS

The significantly higher arsenic concentration in the bacterial enriched fraction supports the hypothesis that the bacteria consortium has a fundamental role in the accumulation of arsenic by the hosting sponge. Previous work (Mayzel et al., 2014) concluded that *T. swinhoei* actively takes up arsenic from the environment. Sponge-associated bacteria in *T. swinhoei* reside in the mesohyl (Magnino et al., 1999), therefore any element the bacteria are exposed to must first pass through the pinacoderm layer (Yahel et al., 2003). Since the arsenic concentration in the associated-bacteria is significantly higher than in the sponge cells, the fate of the accumulated arsenic can be deduced; arsenic from the environment passes through a layer of sponge cells (the pinacoderm) to the bacteria in the mesohyl, were it is stored. Further work is underway to better support this claim.

### BACTERIA GROWTH AND IDENTIFICATION

The OTUs in this work are related to known type stains of free-living marine bacteria. They are also frequently found associated to sponges. However, all but one OTU (TSASRA054) are not typical of arsenic-rich environments. While some OTUs have relatives (genus level) inhabiting arsenic rich environments (elaborated below), the most dominant genus (*Ruegeria*) has never been identified in arsenic rich environments. The only study linking any member of *Ruegeria* to arsenic, presents an isolate which is sensitive to low arsenic concentrations (Nithya and Pandian, 2010). At the family level (*Rhodobacteraceae*) there are only two known members known to tolerate arsenic.

The adaptation to a high arsenic environment present in the sponge indicates the OTUs identified in this work are likely associated-bacteria of *T. swinhoei*. The results of the BLAST search, showing many OTUs are frequently identified from *T. swinhoei* further supports the claim of association. This is a good example in which a phenotype can be used to infer association regardless of 16S rRNA sequence similarity. None of the isolated bacteria is related to bacteria known to dissimilatory reduce arsenate or oxidize arsenite in chemolithoautotrophic pathways. The identified bacteria are all related to heterotrophic bacteria and regarded as arsenic-tolerant.

Two of the eight genera identified in this work (*Aliagarivorans* and *Brevibacterium*) have not been isolated in previous studies of *T. swinhoei* (Lavy et al., 2014), nor were they identified in molecular-based studies (Hentschel et al., 2002; Schmitt et al., 2012). As mentioned above, the OTUs in this work are regarded as likely associated-bacteria, based on the phenotype of arsenic tolerance. The present work shows that the use of selective agents based on *in vivo* measurements can highlight potential roles of associated-bacteria unnoticed before.

Members of the genus *Vibrio*, isolated on three types of culturing media, are commonly cultured from arsenic-contaminated environments (Takeuchi et al., 2007; Shakya et al., 2012), but the OTUs in the current study are not related to known arsenic-resistant strains. TSASRA037, for example, shares 98% 16S rRNA gene sequence similarity with *Vibrio harveyi*, which is used as an indicator of arsenic pollution because of its low tolerance to this element (Thomulka et al., 1993).

Two other genera typically found in arsenic rich environments are *Micrococcus* and *Pseudomonas* (Takeuchi et al., 2007; Fan et al., 2008; Quemeneur et al., 2010; Liao et al., 2011; Shakya et al., 2012). In this study only one of the three OTUs attributed to the genera above was previously identified in such environments. TSASRA054 is closely related to *Pseudomonas xanthomarina*, identified in clone library from arsenic contaminated proglacial soil (Srinivas et al., 2011). The two OTUs of *Pseudomonas* (TSASRA007 and TSASRA054) can tolerate up to 50 and 10 mM arsenate, respectively. *Pseudomonas* spp. are highly sought after as bioremediators of arsenic-polluted waters (Zawadzka et al., 2007; Mondal et al., 2008). Arsenic-contaminated drinking water is a worldwide problem affecting the health of millions of people, especially in under-developed countries (Mandal and Suzuki, 2002; Belluck et al., 2003; Han et al., 2003; Sun et al., 2006; Brinkel et al., 2009; Pal et al., 2009). Contamination of seafood is another major threat (Ahmed et al., 1993; Neff, 1997; Mason et al., 2000; Kirby and Maher, 2002; Rahman et al., 2012). Therefore, the development of cheap, easy to use methods for the bioremediation of water is of utmost importance.

The use of a modular design for culturing media was beneficial for elucidating the key parameters, essential for a bacterium’s culture. Three OTUs grew on two types of media and in each case the media shared a selective parameter. TSASRA003 and TSASRA037 grew only on media supplemented with arsenate (AsVOC and AsVanS). These findings may suggest that TSASRA003 and TSASRA037 are only tolerant of arsenate (at the tested concentrations). TSASRA019 could tolerate both forms of arsenic, but only under microaerophilic conditions with sulfide or nitrate (**Table 2**). This implies TSASRA019 relies on supplemented nutrients for growth in microaerophlic conditions and may be susceptive to the carbohydrates used in this work. Nitrate was also supplemented to AsIIIOC and AsIIIanC, but TSASRA019 did not grow on this media.

Under microaerophilic conditions, bacteria grew on medium AsVanS, and to a lesser extent on AsIIIanN. In both cases, the media were not supplemented with organic carbon, apart from the impurities in the agar. In contrast, no bacteria grew on anaerobic media with supplementation of carbohydrates (AsVanC and AsIIIanC) or when no nutrients were added (AsIIIanP). It thus appears that under microaerophilic conditions nutrients such as sulfide and nitrate are more important than supplementary organic carbon. These nutrients can serve as electron acceptor/donor in anaerobic respiratory electron transfer. Modular media design reveals its virtues in assisting to deduce bacterial metabolic needs when comparing AsVOC and AsVanC. While the former medium enabled the growth of 8 OTUs (38 isolates), no bacteria was found on AsVanC. It is clear that depriving oxygen led to loss of ability to grow with only supplemented carbohydrates. On the other hand, microaerophilic conditions alone did not inhibit bacterial growth as demonstrated on medium AsVanS. Two OTUs (TSASRA003 and TSASRA037) were able to grow on both AsVOC and AsVanS, while not growing on AsVanC. From this it can be implied that sulfide has some role in the bacteria’s tolerance to arsenate in microaerophilic conditions. Alternatively, sulfide may not be directly connected to arsenic tolerance but rather supported bacterial growth in some other pathway. It would be interesting to further test OTUs growing on AsVanS, since there are bacteria which couple sulfide oxidation to dissimilatory reduction of arsenate (Hoeft et al., 2004). Even if bacteria growing on AsVanS can use arsenate in the respiratory electron transfer chain, their ability to grow without arsenate in the arsenate-tolerance assay indicates that such a pathway is not obligatory.

### ARSENATE-TOLERANCE ASSAY

The majority of OTUs tested in this work tolerated arsenate from 5 to 50 mM. All tested isolates grew in the presence of 5–10 mM arsenate. This concentration is equivalent to 6150–12300 mg/Kg arsenic (dry weight), the arsenic concentration measured in the cell-enriched fractions (**Figure 1**). The tolerance of the bacteria to the arsenic concentration found in their source environment further supports the claim that these bacteria are sponge-associated.

An interesting observation was that most OTUs did not grow on medium AsVOC without the presence of arsenate. The affect of arsenate on pH provides the most likely explanation for the low growth. Arsenate, like its homolog phosphate, has a buffering capacity. Without arsenate AsVOC is more acidic, due to the supplemented carbohydrates. This is not the case for medium AsVanS that contains bicarbonate, a strong buffer. This is evident when comparing the growth of TSASRA003 and TSASRA037. These OTUs were tested on both media but grew only on AsVanS, at 0 mM arsenate.

Two growth patterns were observed in the assay, declining or unaffected growth. In the first, growth of bacteria was inhibited at high arsenic concentration. In the latter, bacterial grew on all concentrations. Only members of *Gammaproteobacteria* showed unaffected growth, but also contained the least tolerant OTU (TSASRA054). All *Vibrio* OTUs tolerated the maximum arsenate concentration tested in this work (100 mM), with no apparent affect on their growth. The only other OTU that tolerated 100 mM arsenate was of the genus *Aliagarivorans* (TSASRA084).

Different OTUs in the present work have a different tolerance level to arsenic. The least tolerant OTU can tolerate 10 mM arsenate while the most tolerant OTUs can withstand 100 mM arsenate with no apparent effect. The deferential arsenic tolerance of the sponge-associated bacteria in this work leads to the hypothesis that there may be spatial gradients of arsenic within the sponge body. Further examination for spatial differences in the sponge body should follow to test this hypothesis.

### PRECIPITATION OF ARSENIC

Precipitation of arsenic is a known mechanism of arsenic resistance. While the most studied cases of bacterial arsenic mineralization are those of sulfur reducing and iron oxidizing bacteria, under anaerobic conditions, the precipitation of arsenic with magnesium is also assumed (Slyemi and Bonnefoy, 2012). Of approximately 60 minerals containing both arsenic and magnesium only two minerals have a 3:1 Mg:As ratio (Robins, 1981; Anthony et al., 2003; Ralph and Chau, 2007). Of these, only one mineral – hoernesite [Mg_3_(AsO_4_)_2_], is insoluble in water. Hoernesite is naturally found as a secondary mineral occurring in thermally metamorphosed limestone (Anthony et al., 2003; Ralph and Chau, 2007) and in arsenic contaminated soils near toxic waste sites (Magalhaes, 2002). However, the OTUs isolated from *T. swinhoei* precipitated arsenic with magnesium under aerobic conditions and as a primary mineral. The crystallization of hoernesite occurs only in environments rich in both arsenic and magnesium (Magalhaes, 2002). Although magnesium is one of the major elements of ASW (1284 ppm), sulfur (as sulfate) concentration is twice that of magnesium (2712 ppm). Arsenic is also to abiotically precipitate mainly with sulfur (Realgar, Orpiment), iron (Scorodite), or both (Arsenopyrite; Smedley and Kinniburgh, 2002). Therefore, it was to be expected that abiotic precipitation of arsenic would occur with sulfur and not magnesium. Considering all of the above, the formation of an uncommon magnesium–arsenic mineral is not likely to occur by abiotic means at the ambient conditions of the growth system. Thus, it is concluded that the precipitation of this unique mineral biologically induced by the bacteria.

Two OTUs, TSARA003 and TSARA026, were found to precipitate a magnesium–arsenic salt. TSARA003 is one of five OTUs for which a hit with >97% sequence similarity was found, with the “*Theonella*” Entrez query during the BLAST search. For TSARA026 hits at the genus level were found with the “*Theonella*” Entrez query. TSASRA003 (*Ruegeria*) was the most dominant OTU in this work. Seventy one percent of isolates on AsVOC and 38.5% of isolates on AsVanS were grouped to this OTU. TSASRA026 can tolerate higher arsenate concentration of than TSARA003. The ability of OTUs to detoxify arsenic by way of mineralization marks them as potentially important contributors to the arsenic accumulation process in *T. swinhoei*. Biomineralization of the toxic and soluble arsenate to an inert mineral, presents a scenario in which bioaccumulation is possible without detrimental effect to the sponge host or its associated-bacteria. Even if these OTUs are present in small numbers in the sponge, mineralization can lead to accumulation of large amounts of arsenic within the sponge overt time. This is also supported by the finding that the arsenic is localized to the bacterial fraction of *T. swinhoei*.

Biomineralization of a unique magnesium–arsenic mineral in two unrelated bacteria raises the following question: is there a common pathway controlling the biomineralization of arsenic in sponge-associated bacteria? One possible option is that the formation of the mineral is mediated by bacteria through creation of a chemo-physical environment, favoring the precipitation of arsenic with magnesium. In this case bacteria do not control the formation of the mineral, but rather form an area enriched with both arsenate and magnesium. A more intriguing option is that bacteria control the formation of the mineral by some, yet unknown, biological pathway. To date, there is no known pathway related to the precipitation of arsenic (Slyemi and Bonnefoy, 2012) and this warrants further research.

The observation of bacteria capable of immobilizing arsenic holds great potential for further research into the development of an affordable solution to arsenic freshwater pollution by bioremediation. Easily culturable bacterial strains, such as TSASRA003 and TSASRA026, which precipitate arsenic in ambient conditions, may prove useful to that extent.

## CONCLUSION

This study presents culturable arsenic-tolerant bacteria, harbored by *T. swinhoei*. The work also provides evidence for the role of some *T. swinhoei*-associated bacteria in the accumulation process of arsenic. The enriched bacterial fraction contained higher amounts of arsenic than the sponge enriched fraction and the majority of cultured OTUs showed high tolerance to arsenic. A third of isolated OTUs were tolerant to concentrations an order of magnitude higher than measured in the bacterial cell fraction. Two OTUs mineralized arsenate to an inert mineral that can be stored with no toxic effects, thus providing a plausible mechanism for the accumulation of arsenic in *T. swinhoei*. Even though based on 16S rRNA sequence similarity all the bacteria identified in the present work are genetically related to known free-living bacteria, their high tolerance to arsenic distinguishes them, and further supports the hypothesis that these bacteria adapted to the arsenic-rich internal environment of the sponge host.

Sponges are exposed to metal pollutants in the surrounding water and the continued study of the role played by sponge-associated bacteria in their host’s response mechanisms to such metals may yield new insights regarding the important function of sponges in maintaining the equilibrium of our planet’s oceans.

## Conflict of Interest Statement

The authors declare that the research was conducted in the absence of any commercial or financial relationships that could be construed as a potential conflict of interest.
